# Condorcet and borda count fusion method for ligand-based virtual screening

**DOI:** 10.1186/1758-2946-6-19

**Published:** 2014-05-03

**Authors:** Ali Ahmed, Faisal Saeed, Naomie Salim, Ammar Abdo

**Affiliations:** 1 Soft Computing Research Group, Faculty of Computing, Universiti Teknologi Malaysia, Skudai 81310, Malaysia; 2 Faculty of Engineering, Karary University, Khartoum 12304, Sudan; 3 Computer Science Department, Hodeidah University, Hodeidah, Yemen

**Keywords:** Similarity searching, Virtual screening, Similarity coefficients, Data fusion

## Abstract

**Background:**

It is known that any individual similarity measure will not always give the best recall of active molecule structure for all types of activity classes. Recently, the effectiveness of ligand-based virtual screening approaches can be enhanced by using data fusion. Data fusion can be implemented using two different approaches: group fusion and similarity fusion. Similarity fusion involves searching using multiple similarity measures. The similarity scores, or ranking, for each similarity measure are combined to obtain the final ranking of the compounds in the database.

**Results:**

The Condorcet fusion method was examined. This approach combines the outputs of similarity searches from eleven association and distance similarity coefficients, and then the winner measure for each class of molecules, based on Condorcet fusion, was chosen to be the best method of searching. The recall of retrieved active molecules at top 5% and significant test are used to evaluate our proposed method. The MDL drug data report (MDDR), maximum unbiased validation (MUV) and Directory of Useful Decoys (DUD) data sets were used for experiments and were represented by 2D fingerprints.

**Conclusions:**

Simulated virtual screening experiments with the standard two data sets show that the use of Condorcet fusion provides a very simple way of improving the ligand-based virtual screening, especially when the active molecules being sought have a lowest degree of structural heterogeneity. However, the effectiveness of the Condorcet fusion was increased slightly when structural sets of high diversity activities were being sought.

## Background

Virtual screening refers to the use of a computer-based method to process compounds from a library or database of compounds in order to identify and select the ones that are likely to possess a desired biological activity, such as the ability to inhibit the action of a particular therapeutic target. The selection of molecules with a virtual screening algorithm should yield a higher proportion of active compounds, as assessed by experiment, relative to a random selection of the same number of molecules [1,2].

Many virtual screening (VS) approaches have been implemented for searching chemical databases, such as substructure search, similarity, docking and QSAR. Of these, similarity searching is the simplest, and one of the most widely-used techniques, for ligand-based virtual screening (LBVS) [3]. Similarity search aims to search and scan a chemical database to identify those molecules that are most similar to a user-defined reference structure using some quantitative measures of intermolecular structural similarity [4-8].

There are many different ways to implement the similarity searching based on different similarity models. However, as Sheridan and Kearsley [9] noted, it is most unlikely that a single search mechanism could be expected to perform at a consistently high level under all circumstances. Instead, a more realistic approach to enhancing the effectiveness of ligand-based virtual screening approaches is the use of data fusion [10] or consensus scoring in the structure-based virtual screening literature [11]. Data fusion was first used for similarity searching in the late-Nineties [12-14]. Recently, data fusion has been used to combine the results of the structure and ligand-based approaches to virtual screening [15], their results outperforming any single method in ranking of activities. The latest reviews on using fusion in ligand-based virtual screening can be found in [16,17].

There are two main approaches to data fusion: similarity fusion and group fusion [10,18]. The first type combines ranking from single searches based on multiple similarity measures, while the second one combines ranking from multiple searches based on a single similarity measure. The basic procedure that has been developed for the fusion process is shown in algorithmic form as described below:

The basic procedure for data fusion:

for x = 1:n

for y = 1:N

Use x-th similarity or scoring measure to calculate similarity or score, Simx(qy) for y-th database-structure.

for 1:N

Use the fusion rule to combine the set of n score Simx(qy) for y-th database-structure to give its fused score FSimy,

Sort the database into decreasing order of fused score FSimy.

In this algorithm, there are *n* different similarity measures for calculating the similarity *SIMx(dy)* for each of the *N* structures in the database that is being searched (1 ≤ *x* ≤ *n*, and 1 ≤ *y* ≤ *N*).

The idea of voting algorithms emerged in the 18th century to address the shortcomings of simple majority voting when there are more than two candidates. According to Montague and Aslam [19] and Riker [20], there are two main voting algorithms: majoritarian and positional voting algorithms. Majoritarian voting algorithms are based on a series of pairwise comparisons of candidates, while positional algorithms are based on the ranking a candidate receives.

In this paper, the authors examined the use of Condorcet fusion in order to improve the effectiveness of ligand virtual screening by enhancing the recall of active compound structure. In our proposed model, for each similarity measure the top retrieved structures represent the voters; each candidate’s similarity measures received a number of points or votes depends on the similarity values of the retrieved structures. At last, Borda’s count method evaluated by summation of these points to find the winner candidate’s measure. The winner candidate got the highest number of points.

## Methods

This study has compared the retrieval results obtained using two different similarity-based screening models. The first screening system was based on the Tanimoto (TAN) coefficient, which has been used in ligand-based virtual screening for many years and is now considered a reference standard. The second model, the proposed model of this study, was based on the Condorcet model proposed by Montague and Aslam [19]. In our approach, the two groups of similarity measures were used, the first group is seven of the association coefficients: Jaccard/Tanimoto, Ochiai/Cosine, Sokal/Sneath(1), Kulczynski(2), Forbes, Fossum and Simpson; the second group is four of the distance coefficients: Mean Euclidean, Mean Canberra, Divergence and Bray/Curtis. The results from the two groups were used together and the Condocert fusion based on combining ranking from single searches for each of the eleven similarity measures is achieved. More details about the above similarity measures or metrics found in the early study proposed by Ellis et al. [21].

### Tanimoto-based similarity model

This model used the continuous form of the Tanimoto coefficient, which is applicable to the non-binary data of the fingerprint. *S*_*K,L*_ is the similarity between objects or molecules *K* and *L*, which, using Tanimoto, is given by Equation 1:

(1) $$S_{\mathrm{SK}}=\frac{\sum_{j=1}^{M}\left(w_{\mathrm{jk}}w_{\mathrm{jl}}\right)}{\sum_{j=1}^{M}{\left(w_{\mathrm{jk}}\right)}^{2}+\sum_{j=1}^{M}{\left(w_{\mathrm{jl}}\right)}^{2}-\sum_{j=1}^{M}\left(w_{\mathrm{jk}}w_{\mathrm{jl}}\right)}$$

For molecules described by continuous variables, the molecular space is defined by an *M* × *N* matrix, where entry *w*_*ji*_ is the value of the *j*^*th*^ fragments (1 ≤ *j* ≤ *M*) in the *i*^*th*^ molecule (1 ≤ *i* ≤ *N*). The origins of this coefficient can be found in [21].

### Condorcet-based fusion model

In this study we start our search using single reference structure and then the retrieved results based on different values of *n* represent the input of this process, which we will call a voting profile. Depending on the numbers of points, a social choice function based on Borda count that uses the positional voting procedure and Condorcet voting algorithm that uses majoritarian method will map voting profiles to a set of candidates — the winners.

The Borda count is perhaps the most sensible positional voting procedure. In the Borda count implemented here, for each voter, each candidate receives *n* points (*n* is the number of points in the retrieved structures in top-n results). The pairwise comparisons of candidates, based on the Condorcet voting algorithm that uses the majoritarian method, select the winner similarity method with the most points received. This process is repeated for each activity class.

In this method, eleven similarity measures and four different values of top retrieved structures were examined. The retrieved structures in each top retrieved represent the voter population to elect the winner similarity measures based on the Borda count method of points achieved by each candidate measure. The Condorcet-based fusion algorithm is described as follows:

Condorcet-based Fusion Algorithm

1. for z = 1 top-n % n is number of activity classes in the data set

2. get the top-n ranking score for the each similarity measure

3. for x = 1 to m do % m is number of similarity measures

4. Assign value to each similarity measure equal to the a number of votes or points in retrieved topn structures in the results

5. find out the total Borda score for each similarity measure,

$$B_{c}=\sum_{i=1}^{\mathrm{topn}}B_{i}$$

% Bi is the number of points for this activity class use the x-th similarity measure in topn for y-th database structure

6. Select the winner similarity measure (Fsimx) using pairwise comparisons based on Condorcet voting algorithm that used majoritarian method

7. end for

8. end for

The complexity of the algorithm is calculated and it processes in a worst time of O(**n**(2 **m** + **top-n**)). This time was calculated based on the following: (i) the outer loop (line 1) is based on the number of activity classes in the data set; thus, the maximum number of iterations is **n**, (ii) the first inner loop (line 3) is also based on the number of similarity measures; the maximum number of iterations is **m**, (iii) the second inner loop (line 5) is based on the Borda score for each similarity measure; the maximum number of iterations is **top-n**. Finally, for the final inner loop (line 6), on Condorcet voting algorithm, the maximum number of iterations is (**m**).

## Experimental design

The searches were carried out using the most popular chemoinformatics databases, the MDL Drug Data Report (MDDR) [22], maximum unbiased validation (MUV) [23] and Directory of Useful Decoys (DUD) [24]. All the molecules in both databases were converted to Pipeline Pilot ECFC_4 (extended connectivity fingerprints and folded to size 1024 bits) [25]; MDDR and MUV data sets have been used recently by our research group in this research area [26-29]. Mathworks Matlab R2012b (UTM license) was used for coding our proposed algorithms; all calculations were run on 2.80 GHz Intel(R) Xeon(R) processors.

The algorithm is started by searching using a single reference structure and the eleven similarity measures (each structure from each activity class); the retrieved output, based on different values of top results, is evaluated. For each retrieved structure per each class, the similarity measure with maximum similarity value receive a high vote or point (the number eleven is given for the best measure and the number one for the worst), then the summation of the Borda scores or vote was calculated by summation of the votes or points for this top retrieved value and the winning measure is the one that has the highest votes or points. Finally, the search was carried out again using the winner measure and the final results were calculated. The explanation example in Table 1 shows the voting profile example of three top retrieved structures, showing that measure S3 is the winner (with 30 votes or points). In this study, the same scenario is used for different numbers of top retrieved or nearest neighbours (10, 20, 50 and 100).

**Table 1:** explanation example on electing winner measure based achieved votes or points

	**Similarity measures**										
	**S1**	**S2**	**S3**	**S4**	**S5**	**S6**	**S7**	**S8**	**S9**	**S10**	**S11**
**Votes or points**	11	9	10	1	3	7	6	4	8	2	5
	9	8	11	4	5	3	1	2	7	6	10
	7	3	9	1	8	4	11	6	2	5	10

For the screening experiments, two data sets (MDDR1 and MDDR2) with 102516 molecules were chosen from the MDDR database. The MDDR1 data set contains 10 homogeneous activity classes and the MDDR2 data set contains 10 heterogeneous activity classes (i.e. structurally diverse). Details of these two data sets are given in Tables 2 and 3. Each row of a table contains an activity class, the number of molecules belonging to the class, and the class’s diversity, which was computed as the mean pairwise Tanimoto similarity calculated across all pairs of molecules in the class using ECFC_4. The second data set, (MUV) as shown in Table 4, was reported by Rohrer and Baumann [23]. This data set contains 17 activity classes, with each class containing up to 30 actives and 15,000 inactives. The diversity of the class for this dataset shows that it contains high diversity or more heterogeneous activity classes. This data set was also used in the previous study by our research group [30].

**Table 2:** MDDR1 structure activity classes

**Activity index**	**Activity class**	**Active molecules**	**Pair-wise similarity**
07707	Adenosine (A1) agonists	207	0.424
07708	Adenosine (A2) agonists	156	0.484
31420	Renin inhibitors 1	1300	0.584
42710	CCK agonists	111	0.596
64100	Monocyclic -lactams	1346	0.512
64200	Cephalosporins	113	0.503
64220	Carbacephems	1051	0.414
64500	Carbapenems	126	0.444
64350	Tribactams	388	0.673
75755	Vitamin D analogous	455	0.569

**Table 3:** MDDR2 structure activity classes

**Activity index**	**Activity class**	**Active molecules**	**Pair-wise similarity**
09249	Muscarinic (M1) agonists	900	0.257
12455	NMDA receptor antagonists	1400	0.311
12464	Nitric oxide synthase inhibitors	505	0.237
31281	Dopamine-hydroxylase inhibitors	106	0.324
43210	Aldose reductase inhibitors	957	0.370
71522	Reverse transcriptase inhibitors	700	0.311
75721	Aromatase inhibitors	636	0.318
78331	Cyclooxygenase inhibitors	636	0.382
78348	Phospholipase A2 inhibitors	617	0.291
78351	Lipoxygenase inhibitors	2111	0.365

**Table 4:** MUV structure activity classes

**Activity index**	**Activity class**	**Pair-wise similarity**
466	S1P1 rec. (agonists)	0.445
548	PKA (inhibitors)	0.430
600	SF1 (inhibitors)	0.445
644	Rho-Kinase2 (inhibitors)	0.416
652	HIV RT-RNase (inhibitors)	0.398
689	Eph rec. A4 (inhibitors)	0.449
692	SF1 (agonists)	0.365
712	HSP 90 (inhibitors) 30	0.413
713	ER-a-Coact. Bind. (inhibitors)	0.389
733	ER-b-Coact. Bind. (inhibitors)	0.352
737	ER-a-Coact. Bind. (potentiators)	0.502
810	FAK (inhibitors)	0.425
832	Cathepsin G (inhibitors)	0.435
846	FXIa (inhibitors)	0.532
852	FXIIa (inhibitors)	0.492
858	D1 rec. (allosteric modulators)	0.400
859	M1 rec. (allosteric inhibitors)	0.386

The third data set used in this study is Directory of Useful Decoys (DUD), this data set has recently been compiled as a benchmark data set, specifically for docking methods. It was introduced by [24] and was used recently in molecular virtual screening [31] as well as molecular docking [32]. The decoys for each target were chosen specifically to fulfil a number of criteria to make them relevant and as unbiased as possible. In this study twelve subsets of DUD with 704 active compounds and 25,828 decoys were used as shown in Table 5.

**Table 5:** **Number of active and inactive compounds for twelve DUD sub datasets, where N**_**a **_**: number of active compounds, N**_**dec **_**: number of decoys**

**No.**	**Data set**	**Active and inactive compounds**	
		**N**_**a**_	**N**_**dec**_
1	FGFR1	120	4550
2	FXA	146	5745
3	GART	40	879
4	GPB	52	2140
5	GR	78	2947
6	HIVPR	62	2038
7	HIVRT	43	1519
8	HMGA	35	1480
9	HSP90	37	979
10	MR	15	636
11	NA	49	1874
12	PR	27	1041
**Total**		704	25828

Searches were carried out using single reference structures and a total of eleven similarity measures. Different numbers of top retrieved or nearest neighbours—10, 20, 50, and 100—were selected (as voter committee or population) for each activity class and used as input to the fusion stage to determine the winner candidate similarity measure. Finally, a search was carried out again based on the winner or fused similarity measure.

## Results and discussion

The results of the searches of MDDR1, MDDR2, MUV and DUD are presented in Tables 6, 7, 8 and 9 respectively, using cut offs at 5%. In these tables, the second column from the left contains the results for the TAN, the third to sixth columns contain the corresponding results when the Condorcet fusion model is used based on different four top retrieved values. Each row in the tables lists the recall for the top 5% for each activity class. The mean rows in the tables correspond to the mean when averaged over all activity classes, and the CI rows represent the 95% confidence interval. The similarity method with the best recall rate in each row is shown as (*), and the best mean recall value is boldfaced. The bottom row in a table corresponds to the total number of (*) cells for each similarity method across the full set of activity classes.

**Table 6:** Retrieval results of top 5% for data set MDDR1

**Activity index**		**TAN**	**Top10**	**Top20**	**Top50**	**Top100**
07707		71.56	87.72	85.62	86.03	85.67
07708		56.96	80.46	92.15	92.25	96.85
31420		88.89	89.07	88.61	89.39	89.45
42710		88.65	90.03	89.66	89.85	90.06
64100		94.85	94.19	94.29	99.31	99.26
64200		78.41	91.69	91.26	91.62	96.94
64220		53.40	70.11	62.54	81.61	89.75
64500		45.28	74.17	74.72	89.06	88.64
64350		92.27	92.52	99.95	97.82	98.28
75755		95.27	95.52	96.61	97.55	95.16
**Mean**		76.55	86.55	87.54	91.45	**93.01**
**CI**	**Lower**	45.28	70.11	62.54	81.61	85.67
	**Upper**	95.27	95.52	99.95	99.31	99.26
Star (*) cells		0	1	1	3	5

**Table 7:** Retrieval results of top 5% for data set MDDR2

**Activity index**		**TAN**	**Top10**	**Top20**	**Top50**	**Top100**
09249		24.64	23.88	24.01	24.64	23.89
12455		11.24	21.26	21.40	13.47	14.17
12464		16.25	23.72	23.60	23.64	37.29
31281		30.57	39.70	32.74	39.54	50.63
43210		16.82	18.45	26.49	29.36	14.17
71522		13.22	16.16	15.87	16.12	16.22
75721		25.81	28.29	27.89	48.22	31.36
78331		16.05	17.80	18.67	18.33	24.76
78348		25.16	26.20	26.29	20.97	26.92
78351		12.70	14.46	14.60	14.85	15.15
**Mean**		19.25	22.99	23.16	24.91	**25.46**
**CI**	**Lower**	11.24	14.46	14.60	13.47	14.17
	**Upper**	30.57	39.70	32.74	48.22	50.63
Star (*) cells		1	0	1	3	6

**Table 8:** Retrieval results of top 5% for data set MUV

**Activity index**		**TAN**	**Top10**	**Top20**	**Top50**	**Top100**
466		6.72	9.05	7.97	8.69	8.90
548		20.85	25.43	26.06	25.82	26.29
600		9.94	13.82	13.85	13.99	15.69
644		23.07	22.47	23.27	22.93	22.85
652		7.74	11.21	10.88	10.52	11.17
689		12.40	13.35	14.76	16.39	12.38
692		7.25	9.28	10.23	6.80	7.76
712		15.63	13.11	13.42	13.62	12.94
713		6.99	9.60	10.37	9.71	11.97
733		9.13	11.60	11.00	12.75	13.31
737		8.50	10.62	10.71	13.36	13.53
810		8.51	9.27	9.37	9.40	10.93
832		18.75	14.23	15.10	15.20	17.38
846		24.56	29.64	30.37	29.88	29.72
852		15.71	24.50	24.48	23.63	23.81
858		7.38	8.29	7.53	8.39	8.47
859		9.51	9.92	9.60	11.32	11.44
**Mean**		12.51	14.43	14.65	14.85	**15.21**
**CI**	**Lower**	6.72	8.29	7.53	6.80	7.76
	**Upper**	24.56	29.64	30.37	29.88	29.72
Star (*) cells		2	3	3	1	8

**Table 9:** Retrieval results of top 5% for data set DUD

**Data set**		**TAN**	**Top10**	**Top20**	**Top50**	**Top100**
FGFR1		7.25	6.08	6.08	7.08	12.08
FXA		8.70	9.52	9.52	9.52	9.52
GART		21.25	21.5	21.5	21.5	7.25
GPB		27.12	29.04	29.04	29.04	35
GR		6.79	6.79	6.79	8.97	13.21
HIVPR		12.74	6.45	6.45	12.9	6.45
HIVRT		4.42	8.42	9.42	4.42	8.42
HMGA		10.02	10.30	15.21	15.35	17.44
HSP90		9.46	9.46	9.46	9.46	8.92
MR		8.67	8.67	8.67	8.67	8.67
NA		6.53	6.53	6.53	6.73	10.73
PR		5.93	7.70	9.70	10.7	12.7
**Mean**		10.74	10.87	11.53	12.03	**12.53**
**CI**	**Lower**	4.42	6.08	6.08	4.42	6.45
	**Upper**	27.12	29.04	29.04	29.04	35.00
Star (*) cells		3	3	4	5	8

A look at the recall values in Tables 6, 7, 8 and 9 enables comparisons to be made between the effectiveness of the various search models. However, a more quantitative approach is possible using the Kendall W test of concordance [33]. This test shows whether a set of judges make comparable judgments about the ranking of a set of objects. Here, the activity classes were considered the judges and the recall rates of the various search models, the objects.

The outputs of this test are the value of the Kendall coefficient and the associated significance level, which indicates whether the value of the coefficient could have occurred by chance. If the value is significant (for which we used cutoff values of 0.05), then it is possible to give an overall ranking of the objects that have been ranked.

The results of the Kendall analyses for MDDR1, MDDR2, MUV and DUD are reported in Table 10 which describes the top 5% rankings for the various searching approaches. In this Table, the columns show the data set type, the value of the Kendall’s coefficient of Concordance (W), the associated probability (p) and the ranks of each of the different searching methods. Table 10 shows that the values of Kendall coefficients vary from 0.441 (agreement is 44.1%) for DUD to 0.594 (agreement is 59.4%) for MDDR1, while the values of associated probability, (p), are (< 0.01) for all recall percentages of the three data sets. This indicates that these values are significant and it becomes possible to give an overall ranking to the objects (searching approaches). Therefore, the ranking of the search methods for all cases is significant and has not occurred by chance.

**Table 10:** Rankings of similarity approaches based on Kendall W test results: MDDR1, MDDR2, MUV and DUD top 5%

**Data set**	**W**	**P**	**Ranking**
MDDR1	0. 594	0.001	Top100 > Top50 > Top20 > Top10 > TAN
MDDR2	0.479	0.008	Top100 > Top50 > Top20 > Top10 > TAN
MUV	0.447	0.002	Top100 > Top50 > Top20 > Top10 > TAN
DUD	0.441	0.009	Top100 > Top50 > Top20 > Top10 > TAN

Using Kendall W may result, in some cases, in the occurrence of tied values for ranking. The effect of ties is to reduce the value of W; however, this effect is small unless there are a large number of ties. When tied values occur, they are each given the average of the ranks that would have been given had no ties occurred and correction factors will be calculated as shown in the following equation:

$$T_{j}=\sum_{i=1}^{g_{i}}\left(t_{i}^{3}-t_{i}\right)$$

where t_i_ is the number of tied ranks in the i*th* group of tied ranks and g_j_ is the number of groups of ties in the set of ranks (ranging from 1 to *n*) for judge *j*. Thus, T_j_ is the correction factor required for the set of ranks for judge *j*, i.e. the j*th* set of ranks.

Some of the activity classes, such as low-diversity activity classes, may contribute disproportionately to the overall value of mean recall. Therefore, using the mean recall value as the evaluation criterion could be impartial in some methods, but not in others. To avoid this bias, the effective performances of the different methods have been further investigated based on the total number of (*) cells for each method across the full set of activity classes. This is shown in the bottom rows of Tables 6, 7, 8 and 9. According to the total number of (*) cells in these tables, Condorcet fusion at Top100 was the best performing search across the three data sets.

The results of the MDDR1 search shown in Table 6 show that Condorcet fusion at Top100 produced the highest mean value compared with other measures. The value of the Kendall coefficient is 0.594. Given that the result is significant, since associated probability is < 0.01, the overall ranking of the different approaches is Top100 > Top50 > Top20 > Top10 > TAN for the cut off 5%, which shows that the proposed method has a high rank value. Similarly, For MDDR2 data set, our proposed method has the highest rank at cut off 5%. On the other hand, the MDDR2 searches are of particular interest, since they involve the most heterogeneous activity classes in the three data sets used, and thus provide a complete test of the effectiveness of a screening method. Table 7 shows that Condorcet fusion at Top100 gives the best performance out of all the methods for this data set at cut off 5%.

While the MDDR1 dataset includes highly similar activities, the MUV and DUD datasets have been carefully designed to include sets of highly dissimilar actives. Most of the similarity methods as well as our proposed method here show a very high recall rate for the low diversity dataset and very low recall for the high diversity datasets, such as MDDR2, MUV and DUD used in this study.

**Figure 1:**
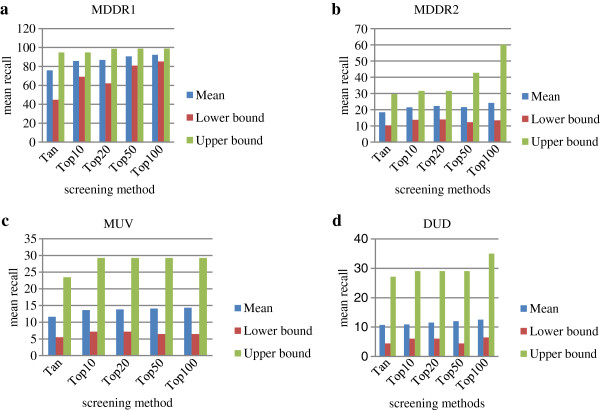
Performance with 95% confidence bound for the three screening methods with a) MDDR1, b) MDDR2, c) MUV and d) DUD data sets at top 5%

Figure 1 showing the mean, lower and upper bounds of the confidence intervals of different methods, revealing that we can be 95% confident that the Condorcet fusion at Top100 method performs best for the three data sets. Therefore, on the basis of these results, we can say with 95% statistical certainty that our proposed method search will do better than conventional similarity systems.

An ROC curve describes the trade-off between sensitivity and specificity, where the sensitivity is defined as the ability of the model to avoid false negatives, and the specificity relates to its ability to avoid false positives.

**Figure 2:**
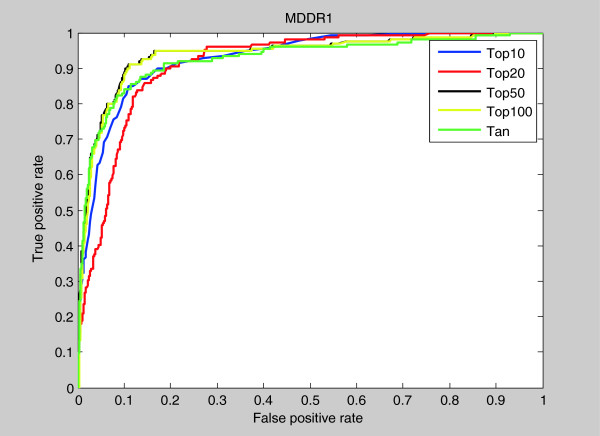
ROC curves and AUCs at 5% cutoff of MDDR1 data set.

The area under the ROC curve (AUC) is a measure of the model’s performance: the closer AUC is to 1, the better is the performance of the prediction. In our study we used the ROC curve to study the performance of different methods at cutoff 5%. Visual inspection of Figure 2 provides a preliminary indication about the quality of each method for data set MDDR1, The area under curve (AUC) metric was calculated for the data MDDR1 and their results added at the end of Figure 2.

In addition, Friedman’s test [34] was performed as another significant test and the result was reported in Table 11. The outputs of this test are the value of the Friedman’s test (p-values) and associated mean ranks of each method. P-values are often coupled to a significance or alpha (α) level, which is also set ahead of time, usually at 0.05 (5%). Thus, if a p-value was found to be less than 0.05, then the result would be considered statistically significant and the null hypothesis would be rejected [35]. Looking at the mean rank and their associated p-values, we can conclude that the Top100 fusion method outperformed the Tanimoto as well as fusion methods at the other top values, with associated values of 0.001, 0.011, 0.002 and 0.017 for the MDDR1, MDDR2, MUV and DUD datasets respectively.

**Table 11:** Rankings of similarity approaches based on friedman’s test results: MDDR1, MDDR2, MUV and DUD top 5%

**Dataset**	**P**	**Mean ranks**				
		**Tan**	**Top10**	**Top20**	**Top50**	**Top100**
**MDDR1**	0.001	1.40	2.90	2.60	4.00	4.10
**MDDR2**	0.011	1.55	3.00	3.10	3.35	4.00
**MUV**	0.002	1.76	2.76	3.29	3.35	3.82
**DUD**	0.017	2.17	2.67	2.96	3.54	3.67

In many fundamental problems, ranging from information retrieval to drug discovery, only the very top of the ranked list of predictions is of any interest and ROCs and AUCs [36,37] are not very useful. New metrics, visualizations and optimization tools are needed to address this “early retrieval” problem [38-40]. In this study, two performance metrics: Enrichment Factor (ER 1%) and Boltzmann-Enhanced Discrimination of ROC (BEDROC) (α = 20) [41] were used as additional and latest powerful performance evaluation metrics and their results were reported in Table 12. Table 12 shows average and median of (EF 1%) and BEDROC (α = 20) enrichment results. The average enrichment using the Top100 fusion method across the 10 targets of MDDR and the 17 MUV targets improves considerably over the best single method. However, the conclusion which can be drawn from Table 12 is the similar to what was derived from Figure 1, Figure 2 and Tables 6, 7, 8, 9, 10 and 11.

**Table 12:** Enrichment values of (BEDROC α = 20) and (EF 1%) using our proposed method on MDDR1, MDDR2, MUV and DUD data sets

	**MDDR1**				**MDDR2**				**MUV**				**DUD**			
	**BEDROC (α = 20)**		**EF (1%)**		**BEDROC (α = 20)**		**EF (1%)**		**BEDROC (α = 20)**		**EF (1%)**		**BEDROC (α = 20)**		**EF (1%)**	
**Method**	**Mean**	**Median**	**Mean**	**Median**	**Mean**	**Median**	**Mean**	**Median**	**Mean**	**Median**	**Mean**	**Median**	**Mean**	**Median**	**Mean**	**Median**
**TAN**	0.49	0.47	80.52	84.99	0.30	0.35	23.25	22.98	0.38	0.36	16.94	16.87	0.17	0.15	20.87	19.55
**Top10**	0.50	0.54	81.85	89.10	0.32	0.36	24.85	23.11	0.40	0.37	17.99	16.64	0.19	0.18	21.67	20.83
**Top20**	0.52	0.49	86.31	89.10	0.35	0.36	23.99	23.79	0.41	0.35	18.70	16.90	0.21	0.16	22.59	20.74
**Top50**	0.55	0.47	86.99	88.55	0.36	0.34	24.72	21.45	0.42	0.39	17.72	17.01	0.29	0.28	23.22	21.05
**Top100**	0.63	0.62	91.17	90.05	0.47	0.51	27.87	23.99	0.45	0.40	19.94	18.64	0.35	0.30	25.61	22.14

Furthermore, our results were compared with recent similar studies such as rank- based group fusion by Chen et al. [42] and standard score (Z-score) by Sastry et al. [39]. In Chen et al. study, the mean recall of their RKP method for MDDR1 data set range from 94.20 to 94.30, while in our method the minimum value of the upper band is 95.27 for Top10 and the maximum value is 99.95 for the Top100 method. Similarly, the best mean recall for the MDDR2 data set of our method is 50.63 for the activity index 31281 compared with 48.98 with their results. In addition, Sastry et al. used the top 1% of MDDR1 and the best mean recall for their method was 43.8 for the RXG combination method, when running our experiment and get 1%, the best mean recall of our method is 44.35 which slightly outperformed their findings.

## Conclusion

In this study, we have developed a Condorcet fusion model to enhance the effectiveness of ligand-based virtual screening. The overall results of our proposed method show that the screening similarity search outperformed the Tanimoto which considered the conventional similarity methods. In addition, there was evidence to suggest that our proposed method, Condorcet fusion at Top100, was more effective for high diversity data sets.

## Competing interests

The authors declare that they have no competing interests.

## Authors’ contributions

AA is a Post-Doctoral researcher and performed the experiments under the supervision of NS and AA. All authors read and approved the final manuscript.

## Authors’ information

Ahmed A: B. Comp. Eng. (Karary University, Sudan), M. Sc. Comp. Sc. (University of Khartoum, Sudan), Ph. D Comp. Sc. (UTM, Malaysia).NS: Professor Dr. B. Comp. Sc. (UTM, Malaysia), M. Sc. Comp. Sc. (W. Michigan, US) Ph. D Info. Sc. (Univ. of Sheffield, UK).
